# Themes for the Thoughtful

**Published:** 1876-06

**Authors:** 


### *THEMES FOR THE THOUGHTFUL.*

---

THE practice is to hang up a quarter of meat by the tendons at the small end. This is convenient, but it rapidly impoverishes the flesh by the gravitation of the rich juices to the large portion, where the unprotected cut surface permits rapid evaporation. Thus little juice remains to facilitate the cooking process and soften the fibers. Dry steaks and flavorless roasts can often be accounted for in this way. The practice should be abandoned.

---

THERE are ten thousand mills and many millions of dollars employed in the United States in robbing our wheat-flour of its real food elements, to please the eye. It is now universally admitted that the process of sifting or bolting out the grey matter of the grain, excludes from our bread nearly all the gluten and phosphate, which are valuable in the blood and muscle and brain and bone-making processes. It is demonstrated that highly nutritive bread can not be very white bread by any possibility. The same is true of other articles of food. Very white squash or cheese or butter is weaker as food, than the naturally golden varieties. A vastly more concentrated food can be made by excluding the portion of the wheat which we now eat as flour, and depending upon the darker part, which is at present employed to give strength and vigor to horses and cattle. A genuine whole-wheat flour is what Americans must have, or the race is certain to degenerate. There are uses in the arts for the starch-portion which we now eat, and if any part of the grain is to be banished from our tables, let it be this.

---

SCIENCE avers that dry food is more digestible, and consequently more useful, for man and other animals, and that copious draughts of fluid should not follow eating. Owners of horses should especially bear this fact in mind. When water is swallowed by these quadrupeds, nearly all of it goes directly to the intestines situated below the stomach. If the bulk of water is considerable, whatever of solids the stomach contains is sure to be washed through the lower orifice. In such cases the food not only affords no nourishment, but acts as a dangerous irritant to the alimentary canal, inducing colic, diarrhœa, and inflammation of the bowels. A very small quantity of fluid may be swallowed directly after eating without disadvantage, but even this should be slightly warm, in order that the tone of the stomach may not be greatly lowered. Human dyspeptics may try this plan with advantage.

---

HOUSE plants do best in rather cool rooms, or at a temperature not so high as sixty degrees. This is the reason we often see finer plants in the cottages of the mechanic and laboring man, than in the warm, furnace-heated mansions of the rich. In the latter, however, much improvement may be made by keeping the air-chamber of the furnace abundantly and constantly supplied with evaporating water—at least eight or ten gallons daily for a medium-sized furnace; and if the plants still appear to suffer from dry air, hang wet napkins over them. If this attention can not be given, the plants should be selected from such sorts as grow naturally in dry climates, such as the cactus, sedum, etc. House plants are often injured by the accumulation of dust in sweeping, which may be partly remedied by syringing, and more easily by placing large funnels of tissue paper inverted over the plants till the dust settles. Tissue paper is best, on account of its little weight on the plants.

---

WILL the United States never enjoy the advantages of a Government sanitary service, similar to that possessed by Great Britain? Millions of money are spent in hunting for stars, while not a dollar goes into the organized search for disease, its origin and means of prevention. Yet knowledge adequate to the saving of one human life among us, is of more value than the discovery of a thousand stars, having their orbits hundreds of miles away. We want medical statistics, and a properly organized system of public hygiene. A medical Signal-Service, as far-reaching as that which now provides us with a knowledge of the approach of storms, and indicates the weather probabilities, would be of immense value, and is entirely feasible. Unhealthy localities would soon be appropriately branded, and the criminally imperfect sanitary conditions of many crowded sections would be pointed out, and possibly remedied under the glare of general publicity.

---

THREE hundred thousand American invalids annually seek health in sanitary resorts. The leading disease is rheumatism, or its kinsman, gout. The sea-side is found to aggravate this class of disorders, as a rule, and high mountain sections also prove unfavorable. A warm climate, with access to warm springs for abundant bathing, is the appropriate resort for this class of sufferers. Malarial disorders find relief in high and dry spots. Debilitated persons, with small nutritive or digestive powers, are wonderfully benefited by sea-living, sea-bathing, and sea-water drinking. The annual average death-rate in cities, is thirty-two per thousand inhabitants; in the country, only twenty-four. This fact demonstrates that city sick-folk should hasten to the country, and that those sick in the country should scrupulously avoid the city, whatever other change of locality they may make.

---

THERE are two animals which seem to be incompatible—sheep and dogs. It has come to be a question as to which farmers shall cultivate, since both can not accupy the same locality at the same time. In Georgia there is now about one dog to every three sheep, or, to be exact, three hundred and nineteen thousand sheep, and one hundred and four thousand dogs. Thus the inhabitants of Georgia have just about mutton enough on the hoof to feed its dogs one month. Yet this army of curs only killed twenty-eight thousand six hundred and twenty-five sheep last year, so carefully were they watched by able-bodied men, at a quarter of a million dollars or so. Strange that Georgia should complain of hard times.

---

FARMERS will find it profitable to adopt certain leading rules, and to steadfastly adhere to them. It will pay them largely, for example, to take good papers and magazines, and to read them; to keep a careful account of all farm operations, contracts entered into, things borrowed or loaned, money received or expended, things contemplated and things done; to see that all implements have a safe and sheltered place, and are confined to it when not in actual use; to repair tools and buildings at the proper time; to see that all fences are in good condition; to plant trees each year, and to care for them intelligently; to see to it that cattle are comfortably housed in bad weather, that no unsound, decayed or mouldy food be given them, and that they are permitted to drink no water less pure than that which is placed upon their owner's table. All these suggestions are in the line of genuine economy and thrift, as well as in the direction of honor and good morals.

---

THE substitution, in dairies, of pans large enough to hold the milk of a large milking for the ordinary small ones now in use, would save at least half the labor expended at present. Such pans should be elevated to about the height of an ordinary table, and should have a hole in the bottom with a spout attached for discharging the skim-milk into a vessel to be cared for by the men folks. Where the whole of one milking is put into a single pan, and the sour milk discharged by simply pulling out a plug from a hole in the bottom of the pan, the entire operation of skimming a mess of milk, and disposing of the sour milk, and rinsing and scalding the pan ready for use again, in a small dairy of eight or ten cows, will not require more than a minute's labor to care for the milk of a cow each morning and evening. In larger dairies it will not require a minute per cow. A farmer's wife, who took the sole care of the milk of forty-nine cows, which make over fifty pounds of butter daily, declares that she can, without haste, skim the milk of one milking, empty the sour milk, and cleanse the pan, and have it ready for use again in twenty minutes, and that it is as easy for her to take care of that number of cows as to wash the dishes for her family. In dairy rooms, where no water is used, the pans should have seventy-five to one hundred square inches surface for the milk of each cow. If the milk is to be cooled with water, fifty to sixty square inches of surface will do.

---

PERSONS suffering from chronic lung-difficulties, are usually benefited by a prolonged stay in localities of great altitude, especially if lacking humidity. Among the most desirable places for consumptives are the following: Sante Fe., N. M., having an altitude of 6,846 feet; Bismarck, Dak., 1,645; Breckenridge, Minn., railroad track, 963; Cheyenne, Wyoming, U. P. track, 6,043; Corsicana, Texas, I. C. track, 425; Denison, Texas, I. C. track, 752; Denver, Col., K. P. track, 5,087; Dodge City, Kansas track, 2,465; Fort Burton, Mon., 2,074 approximate; Fort Scully, Dak., low water Missouri River, 1,512 approximate; North Platte, Neb., railroad track, 3,821; Pembina, Dak., railroad grade, 797; Pike's Peak, Col., 14,147; Salt Lake City, U., 4,260; Virginia City, Mon., 5,482 approximate; Yankton, Dak., railroad track, 1,305.

